# CSF neopterin and quinolinic acid are biomarkers of neuroinflammation and neurotoxicity in FIRES and other infection‐triggered encephalopathy syndromes

**DOI:** 10.1002/acn3.51832

**Published:** 2023-06-20

**Authors:** Russell C. Dale, Terrence Thomas, Shrujna Patel, Velda X. Han, Kavitha Kothur, Christopher Troedson, Sachin Gupta, Deepak Gill, Stephen Malone, Michaela Waak, Sophie Calvert, Gopinath Subramanian, P. Ian Andrews, Tejaswi Kandula, Manoj P. Menezes, Simone Ardern‐Holmes, Shekeeb Mohammad, Sushil Bandodkar, Jingya Yan

**Affiliations:** ^1^ Kids Neuroscience Centre, The Children's Hospital at Westmead, Faculty of Medicine and Health University of Sydney Westmead New South Wales Australia; ^2^ Clinical School, The Children's Hospital at Westmead, Faculty of Medicine and Health University of Sydney Westmead New South Wales Australia; ^3^ Department of Paediatrics, Neurology Service KK Women's and Children's Hospital Singapore Singapore; ^4^ Khoo Teck Puat‐National University Children's Medical Institute National University Health System Singapore Singapore; ^5^ TY Nelson Department of Neurology and Neurosurgery, The Children's Hospital at Westmead The University of Sydney Westmead New South Wales Australia; ^6^ Department of Neuroscience Queensland Children's Hospital South Brisbane Queensland Australia; ^7^ Department of Paediatrics John Hunter Children's Hospital Newcastle New South Wales Australia; ^8^ Department of Neurology Sydney Children's Hospital Network Sydney New South Wales Australia; ^9^ Department of Biochemistry The Children's Hospital at Westmead Westmead New South Wales Australia

## Abstract

**Objective:**

Infection‐triggered encephalopathy syndromes (ITES) are potentially devastating neuroinflammatory conditions. Although some ITES syndromes have recognisable MRI neuroimaging phenotypes, there are otherwise few biomarkers of disease. Early detection to enable immune modulatory treatments could improve outcomes.

**Methods:**

We measured CSF neopterin, quinolinic acid, kynurenine and kynurenine/tryptophan ratio using a liquid chromatography coupled to tandem mass spectrometry (LC–MS/MS) system. The CSF of 18 children with ITES were compared with acute encephalitis (*n* = 20), and three control groups, namely epilepsy (*n* = 20), status epilepticus (*n* = 18) and neurogenetic controls (*n* = 20).

**Results:**

The main ITES phenotypes in 18 patients were acute encephalopathy with biphasic seizures and late restricted diffusion (AESD, *n* = 4), febrile infection‐related epilepsy syndrome (FIRES *n* = 4) and other ITES phenotypes. Influenza A was the most common infectious trigger (*n* = 5), and 50% of patients had a preceding notable neurodevelopmental or family history. CSF neopterin, quinolinic acid and kynurenine were elevated in ITES group compared to the three control groups (all *p* < 0.0002). The ROC (area under curve) for CSF neopterin (99.3%, CI 98.1–100) was significantly better than CSF pleocytosis (87.3% CI 76.4–98.2) (*p* = 0.028). Elevated CSF neopterin could discriminate ITES from other causes of seizures, status epilepticus and febrile status epilepticus (all *p* < 0.0002). The elevated CSF metabolites normalised during longitudinal testing in two patients with FIRES.

**Interpretation:**

CSF neopterin and quinolinic acid are neuroinflammatory and excitotoxic metabolites. This CSF metabolomic inflammatory panel can discriminate ITES from other causes of new onset seizures or status epilepticus, and rapid results (4 h) may facilitate early immune modulatory therapy.

## Introduction

Infection‐triggered encephalopathy syndromes (ITES) are a group of clinical and radiological phenotypes, that are due to a neuroinflammatory response after common childhood infections.^1^ A genetic vulnerability is presumed and can be demonstrated in some patients (such as *RANBP2* in acute necrotising encephalopathy), but the majority of patients with ITES do not harbour genetic variants that are measurable in current clinical practise.^2^

ITES can occur as a recognisable ‘clinico‐radiological phenotype’, such as acute encephalopathy with biphasic seizures and late restricted diffusion (AESD), febrile infection‐related epilepsy syndrome (FIRES), acute necrotising encephalopathy of childhood (ANE), acute fulminant cerebral oedema^3^ (AFCE) and mild encephalopathy with reversible splenial lesion (MERS).^1^, ^4^ Less common ITES syndromes are acute infantile encephalopathy predominantly affecting the frontal lobes (AIEF) which is a variant of AESD,^5^ and hemiplegia hemiconvulsion epilepsy syndrome (HHE).^6^ Given the similarity of FIRES with other ITES phenotypes (fever and infection provocation, acute encephalopathy, distinct clinico‐radiological phenotypes, evidence of neuroinflammation without encephalitis), we present these syndromes all together under the umbrella term of ITES.

Patients with ITES present with an acute febrile encephalopathy, similar to acute encephalitis. Acute encephalitis is due to direct invasion of the central nervous system (CNS) by a neurotropic virus/microorganism with secondary innate and adaptive immune cell inflammation affecting the brain parenchyma (due to T cells, B cells and autoantibodies). Alternatively, acute encephalitis is due to infiltration of the brain by peripheral innate or adaptive immune cells and autoantibodies as part of an autoimmune or inflammatory process. Both viral and autoimmune encephalitis can result in secondary CNS resident immune (glia) activation.^7^

By contrast, in ITES, there is no definable autoimmune process, and no infiltration of the brain by peripheral immune cells. Instead, the inflammation of ITES is thought to be due to an indirect activation of the immune system within the brain (such as microglia and astrocytes), secondary to infection occurring outside of the CNS.^2^ This separation of ITES from encephalitis is important not only from a diagnostic perspective,^7^ but also from a therapeutic perspective. There is now great interest in the use of immune therapies which target innate immune activation molecules, such as interleukin‐1 and interleukin‐6, which are pro‐inflammatory pleiotropic cytokines that are pro‐epileptic as well as pro‐inflammatory. For example, anakinra, an interleukin‐1 receptor antagonist, and tocilizumab, an IL‐6 receptor antagonist, are potentially valuable therapeutic agents for ITES.^8^, ^9^

Despite this progress, there remain major challenges in making a positive diagnosis and discriminating ITES from encephalitis and other causes of new onset seizures (epilepsy) or new onset encephalopathy.

However, some of the syndromes do not have typical radiological biomarkers, such as FIRES,^10^ which is hard to discriminate from acute encephalitis or other causes of refractory epilepsy in the early days of the illness. Likewise, there are a number of ITES which lack a radiological phenotype (normal MRI imaging) but are clearly infection provoked encephalopathy syndromes (including COVID‐19).

Although cerebrospinal fluid (CSF) cytokines are potentially measurable and have been shown to be elevated in patients with ITES and FIRES,^11^ these cytokines are typically only used for research purposes, and due to the batching nature of the platforms, are often not translatable into clinical settings at this time.

Initially using untargeted CSF metabolomics, followed by targeted metabolomics, we have created a validated and reproducible CSF inflammatory metabolite panel for use in clinical service. We have found that CSF neopterin, quinolinic acid, kynurenine and kynurenine/tryptophan ratio to be useful in detecting neuroinflammation.^12^ The panel uses a liquid chromatography coupled to tandem mass spectrometry (LC–MS/MS) platform, and generates results for patient samples in 4 h, with the capability of individualised testing for clinical urgency.^13^

Given the fact that many ITES lack useful biomarkers, we explored the utility of the CSF inflammatory metabolite panel in a cohort of ITES, and compared the findings to controls that are relevant to the differential diagnosis facing clinicians.

## Materials and Methods

### Cohort

Between 2016 and 2022, 18 patients with ITES were seen at the Children's hospital at Westmead (*n* = 12) or referring hospitals (*n* = 6) and had stored frozen CSF available for analysis, as described in detail below. Regarding therapeutics, intravenous methylprednisolone pulses were 30 mg/kg/day for 3–5 days (maximum 1 g), intravenous immunoglobulin was 2 g/kg for 2–5 days for induction dose and 1 g/kg for subsequent doses when given. Anakinra starting dose was 3–5 mg/kg/day subcutaneously (daily) and final dose was up to 10 mg/kg/day, and tocilizumab was 8–12 mg/kg given intravenously once and repeated every 2 weeks as required. Modified Rankin score (MRS) was generated retrospectively by RCD on review of the clinical histories and discussion with treating clinicians.

To compare the findings in ITES patients, we used control groups that are important differential diagnoses for ITES presentations, namely encephalitis (*n* = 20), epilepsy (*n* = 20), status epilepticus (febrile and afebrile) (*n* = 18) and non‐inflammatory and non‐epileptic neurological controls (neurogenetic, *n* = 20). The ages were not significantly different in any control group compared to the ITES group (Mann–Whitney *U* test) (Table 1).

**Table 1:** The number, sex distribution, age (median and range) and specific aetiology of the ITES group (*n* = 18), encephalitis (*n* = 20), epilepsy (*n* = 20), status epilepticus (*n* = 18) and neurogenetic (*n* = 20) control groups.

Group	Subgroup	Number (males)	Median age (range)	Aetiology
Infection‐triggered acute encephalopathy syndrome (*n* = 18, 9 males, median age 4.9, range 0.7–17.3)	AESD	4 (1 M)	1.9 (0.9–2.7)	AESD (*n* = 3), AIEF (*n* = 1)
	FIRES	4 (3 M)	8 (5–17.3)	FIRES (*n* = 4)
	ANE	2 (0 M)	2.8 (0.7–4.9)	ANE (RANBP2 gene positive *n* = 1, sporadic *n* = 1)
	other	3 (2 M)	3.9 (1.4–8.5)	Hemiplegic hemiconvulsion epilepsy syndrome (HHE), acute fulminant cerebral oedema (AFCE), mild encephalopathy with reversible splenium (MERS) (all *n* = 1)
	Infection encephalopathy syndrome	5 (3 M)	8.0 (4.3–12.5)	Influenza (*n* = 2), SAR‐CoV2 (concurrent rhinovirus), *E. coli* and Salmonella typhi (*n* = 1 each)
Encephalitis (*n* = 20, 7 males, median age 5, range 0.1–10)	Infectious	9 (4 M)	4 (0.1–10)	Suspected viral, unknown (*n* = 3), mycoplasma (*n* = 2), HHV6, HSV2, enterovirus, influenza^1^ (all *n* = 1)
	Autoimmune	11 (3 M)	5 (3.9–7.6)	MOG antibody associated ADEM (*n* = 6), seronegative ADEM (*n* = 2), basal ganglia encephalitis, HSV encephalitis induced anti‐NMDAR encephalitis seronegative autoimmune encephalitis (all *n* = 1)
Epilepsy (*n* = 20, 12 males, median age 4.3, 0.4–15.1)	Genetic developmental epileptic encephalopathy	11 (6 M)	4.5 (0.4–14.5)	GLUT1 (*n* = 2), SCN8A (*n* = 2), CHD2 (*n* = 2), SYNGAP1, SCN1A, FOXG1, GRIN1, 16p13 microdeletion (all *n* = 1)
	Generalised genetic epilepsy	5 (3 M)	8.4 (3.8–15.1)	Juvenile absence (*n* = 2), childhood absence refractory (*n* = 2), childhood absence with eyelid myoclonia (*n* = 1)
	Other	4 (3 M)	3.5 (1.5–4.2)	Doose syndrome (*n* = 2), focal cortical dysplasia, cryptogenic refractory epilepsy (both *n* = 1)
Status epilepticus (*n* = 18, 7 males, median age 3.6, 0.3–7.3)	Febrile status epilepticus	9 (3 M)	1.5 (0.3–7.3)	Febrile status epilepticus (*n* = 9)
	Afebrile status epilepticus	9 (4 M)	4.2 (0.6–5.8)	Afebrile status epilepticus (*n* = 9) (non‐epilepsy)
Neurogenetic (*n* = 20, 8 males, median 2.85 (0.5–14)	Genetic movement disorders	8 (3 M)	5.6 (1.5–14)	GCH1 (*n* = 2), TITF1, DYT1, TNPO2, FOXG1, spinocerebellar syndrome, suspected genetic (all *n* = 1)
	Genetic neurodevelopmental disorder	7 (2 M)	2.7 (0.5–5)	Suspected (*n* = 3), 1p duplication, Unbalanced chromosomal translocation 2p and 9p, STXBP1, SCN8A (all *n* = 1)
	Other neurogenetic	5 (3 M)	1.6 (0.6–4.3)	MAP2K2, CMT1A, TCF4, ERCC8, congenital myasthenia (all *n* = 1)

ADEM, acute disseminated encephalomyelitis; AESD, acute encephalopathy with biphasic seizures and late restricted diffusion; AIEF, acute infantile encephalopathy predominantly affecting the frontal lobes; ANE, acute necrotising encephalopathy of childhood; CHD2, chromodomain DNA helicase binding protein 2; CMT1A, Charcot–Marie–Tooth disease type 1A; DYT1, dystonia 1; ERCC8, excision repair cross complementation group 1; FIRES, febrile infection‐related epilepsy syndrome; FOXG1, forkhead box protein G1; GCH1, GTP cyclohydrolase 1; GLUT1, glucose transporter protein type 1; GRIN1, glutamate receptor inontropic N‐methyl‐D‐asparate subunit 1; HHV6, human herpes virus 6; HSV, herpes simplex virus; ITES, infection‐triggered encephalopathy syndromes; MAP2K2, mitogen‐activated protein kinase kinase 2; MOG, myelin oligodendrocyte glycoprotein; NMDAR, N‐methyl D‐aspartate receptor; SCN1A, sodium voltage‐gated channel alpha subunit 1; SCN8A, sodium channel protein type 8 subunit alpha; STXBP1, syntaxin‐binding protein 1; SYNGAP1, synaptic Ras GTPase‐activating protein 1; TCF4, transcription factor 4; TITF1, thyroid‐specific transcription factor‐1; TNPO2, transportin‐2.

^1^ The patient with influenza‐associated encephalitis had MRI inflammatory changes compatible with encephalitis (rather than an ITES).

### CSF metabolomics

All patient CSF was sent to the Children's Hospital at Westmead Neurochemistry laboratory for routine diagnostic services in multiple aliquots. All samples are frozen at −80 degrees within 1 h. An aliquot that had not been previously thawed was used for this research assay. For the ITES, acute encephalitis and status epilepticus, the CSF samples were taken in the first 3 days of admission (as part of initial diagnostic work up) and before immune therapy, but not necessarily before symptomatic therapy (e.g. anti‐epileptic drugs). For epilepsy and neurogenetic control groups, the CSF was taken during the chronic but symptomatic phase of the illness.

The selected metabolites of interest in the panel are associated with neuroinflammation and were reported as statistically significant in an untargeted metabolomics study. The measurement of neopterin and tryptophan‐kynurenine pathway metabolites (tryptophan, kynurenine, quinolinic acid and kynurenine/tryptophan ratio) was conducted in accordance to the method of Yan et al.,^13^ and reference ranges were used, as reported previously.^14^ In brief, human CSF was deproteinised using metaphosphoric acid/ethylenediaminetetraacetic acid solution, vortexed and centrifuged in Nanosep 0.2 μM centrifugal devices. The supernatant was collected and analysed on the Waters ACQUITY UPLC I‐Class System UPLC system coupled to a Xevo TQ‐XS triple quadrupole mass spectrometer. The Acquity UPLC BEH C18 column (2.1 mm × 150 mm 1.7 μm particle size) was used to achieve chromatographic separation of the metabolites and detected on the mass spectrometer using the multiple reaction monitoring mode.

### Statistics

Statistical analyses were performed using GraphPad Prism 8 (GraphPad, San Diego, USA) and R (The R Foundation for Statistical Computing, version 4.2.2). For the metabolomics data, pairwise non‐parametric tests (Mann–Whitney U test) were performed, and GraphPad Prism was used for analyses and figure generation. We compared the inflammation group with the other four groups (encephalitis, epilepsy, status epilepticus and neurogenetic controls), therefore allowing for 16 pairwise tests, the Bonferroni correction was 0.0031. The pROC R package was used to generate ROC curves and areas under the curves (AUCs).^15^ The 95% CI of the AUC was computed using the pROC *ci.auc* function and DeLong method. Comparisons of two correlated (paired) ROC curves were computed using the pROC *roc.test* function and DeLong method.^16^

### Ethics

The Sydney Children's Hospitals Network Ethics Committee approved this study (LNR/14/SCHN/275; 2019/ETH06182), including informed consent from parents and/or guardians, as per ethics protocol.

## Results

### Infection‐triggered encephalopathy syndrome (Table 2)

**Table 2:** Clinical data of patients.

Case	ITES type	Age (years), sex	Preceding personal or family history	Days infection/fever before ITES	Type infection	First symptoms	MRS nadir	Intensive care	Enceph.	Seizures	Status epilepticus	Other neurology	Inpatient length (days)
1	AESD	1.4 f	Prematurity, neonatal RDS	3	Rhinovirus, bocavirus	Seizure	5	+	+	+ (GTC, biphasic)	+	Dystonia, hypertonia	84
2	AESD	2.5 m	–	2	Influenza A	Status epilepticus	5	+	+	+ (biphasic)	+	Dystonic posturing	21
3	AESD	2.7f	Atypical FC (age 2.2 years)	2	Influenza A	Seizure	4	–	+	+ (focal)	–	Dystonia, bulbar	56
4	AIEF	0.9 f	–	2	Rhinovirus, mycoplasma	Drowsy	5	+	+	+ (focal)	–	Dystonia, hypotonia	11
5	FIRES	5 m	ASD traits	6	Sore throat	Seizure	5	+	+	+ (focal and GTC)	+	Dyskinesia, behaviour	128
6	FIRES	8 f	–	5	Nil local.	Seizure	5	+	+	+ (focal and GTC)	+	dysautonomia	180
7	FIRES	8 m	–	4	Nil local.	Seizure	5	+	+	+ (focal and GTC)	+	Tremor, hyperreflexia	55
8	FIRES	17.3 m	–	1	Nil local.	Seizure	5	+	+	+ (focal and GTC)	+	–	70
9	ANEC (*RANBP2*)	0.7 f	Parent *RANBP2* variant (unaff)	2	Rhinovirus	Seizure	5	+	+	+ (focal)	–	Dyskinesia, bulbar	42
10	ANEC	4.9 F	–	2	Influenza A	Drowsy	5	+	+	–	–	Hypotonic, bulbar	42
11	HHE	1.4 f	–	1	Nil local.	Seizure	5	+	+	+ (focal and GTC)	+	Hemiplegia	28
12	MERS	8.5 m	–	1	Nil local.	Seizure	4	–	+	+ (focal)	–	Confusion	9
13	AFCE	1.7 m	–	1	SARS‐CoV2	Drowsy	6	+	+	+ (GTC)	–	Brainstem death	2
14	ITES^1^	4.3 f	Recent return from Pakistan	7	Typhoid fever	Confusion	5	+	+	–	–	Mutism, shock	23
15	ITES^1^	6 f	Dravet syndrome	7	Influenza A	Confusion, drowsy	5	+	+	–	–	Dysautonomia	18
16	ITES^1^	7.3 m	JIA (on methotrexate)	7	Influenza A	Seizure	4	–	+	+ (GTC)	–	Hypotonia, incoordination	14
17	ITES^1^	10.1 m	ASD, ADHD	3	Rhinovirus, SARS‐CoV2	Seizure	5	+	+	+ (GTC)	+	–	4
18	ITES^1^	12.5 m	Diabetic ketoacidosis	1	*E. coli* septicaemia	Coma	5	+	+	–	–	Dysautonomia	14

ADHD, attention deficit hyperactivity disorder; ASD, autistic spectrum disorder; f, female; FC, febrile convulsion; GTC, generalised tonic–clonic; ITES, infection‐triggered encephalopathy syndromes; JIA, Juvenile idiopathic arthritis; m, male; MRS, modified Rankin scale; Nil local., no localising infections to appreciate source of fever; RDS, respiratory distress syndrome.

The grey shade helps to visualise the different subgroups of the cohort.

^1^ With normal neuroimaging.

#### Demographics and previous history

18 children with infection‐associated encephalopathy syndrome (ITES) had available stored CSF for analysis after routine testing. 12 of the 18 had a recognised clinico‐radiological syndrome: Four patients had AESD (including one with AIEF), four patients had FIRES, two patients had ANE and one patient each with HHE, AFCE and MERS. A further five patients had ITES but normal imaging: These patients lacked features of a clinico‐radiological syndrome, all had an encephalopathy syndrome associated with preceding or concurrent infection but lacked features of encephalitis or meningitis.

Nine of the 18 patients were female and the median age was 4.9 (range 0.7–17.3). The AESD patients tended to be younger than the FIRES patients (Table 2).

Nine of the 18 patients had a preceding personal history or notable family history; a neurodevelopmental syndrome was present in six patients, including autism in two and Dravet syndrome (SCN1A positive) in one patient. Notable immune history was present in two patients (JIA and Type 1 diabetes). One patient with ANE had a pathogenic *RANBP2* mutation, as did an unaffected parent.

#### Infection triggers

All patients had preceding and often ongoing fever and/or infection at the time of neurological symptom onset (Table 2). The mean duration of fever was 3 days (1–7), with AESD, ANE, MERS, HHE and AFCE patients tending to have a shorter fever duration than the FIRES patients (Table 2). A specific infectious agent was identified in 12, with a higher yield in AESD, ANEC and ITES, and lower yield in FIRES. Influenza A was found on nasopharyngeal aspirate in five, rhinovirus in three and SARS‐CoV2 in two patients (Table 2). Three patients had two infectious agents identified (Cases 1 and 17). Other notable infectious triggers of ITES included, Salmonella typhi found in blood in resident returning from Pakistan who developed encephalopathy 7 days into typhoid fever illness; *Escherichia coli* septicaemia in a child presenting a week in to an admission with diabetic ketoacidosis who developed encephalopathy in the absence of meningitis or biochemical explanation.

#### Neurological syndrome

Seizures (*n* = 12) and encephalopathy (*n* = 6, drowsy, coma, behavioural change) were the presenting features. The cohort were very neurologically sick with a MRS of 5 in 14 patients, and MRS 4 in three patients. One patient succumbed to overwhelming COVID‐19‐induced encephalopathy, with acute fulminant cerebral oedema (AFCE) and brain death within 24 h of admission. Fifteen of 18 patients required intensive care. Seizures were dominant features in 14 patients and seizure course is described in more detail in the AESD and FIRES patients in Supplementary Material. Status epilepticus occurred in 8 (all 4 FIRES patients, 2 of 4 AESD, the HHE patient and one COVID‐19‐induced ITES patient). Seven patients had a movement disorder, with dystonia and abnormalities of tone occurring in all four AESD patients. Dysautonomia affected three patients.

#### Investigations (Table 3)

**Table 3:** Investigation outcome of patients.

Case	ITES type	FBC (×10^9^)	LFT (IU/L)	CSF cells (/mm^3^)	CSF protein (g/L)	MRI	EEG	Immune treatment(s)	Other treatment(s)	Duration follow‐up (years)	MRS outcome	Deficits
1	AESD	–	AST 193 ALT 183	0	0.37	AESD (Day 5)	Diffuse slow, electrographic seizures	IVMP Day 3	Gabapentin, Levetiracetam, craniotomy	2.6	5	Severe dystonia‐spastic 4 limb CP, blind
2	AESD	–	ALT 50	1 mono	0.3	n. (Day 2) AESD (Day 5)	Diffuse slow	IVMP day 5 IVIG Day 6	Levetiracetam, Phenytoin, Midazolam infusion	4	4	Severe ID, ASD non‐verbal, ADHD
3	AESD	wcc 3.9 PLAT 139	AST 126	1 mono	0.14	n. (Day 2) AESD (Day 6)	Diffuse slow	IVMP Day 2 IVIG Day 3	Levetiracetam, phenytoin	2.9	2	ADHD, emotional dysregulstion
4	AIEF	–	AST 236 ALT 86	0	0.14	n. (Day 2) AIEF (Day 6)	Diffuse slow	IVMP Day 3	Levetiracetam, phenytoin, midazolam infusion	3	3	Moderate GDD/ID
5	FIRES	–	AST 48 GGT58	15 mono	0.61	n. (Day 3) d/r hippoc. (Day 7)	Slow, multifocal seizures, PLEDs	IVMP Day 6 IVIG Day 6, anakinra Day 8, toci Day 14, rituximab Day 50	Midazolam infusion, ketamine, propofol, multiple AEDs	0.7	3	Moderate GDD/ID, control. epilepsy
6	FIRES	–	GGT 5000	15 mono	0.27	n. (Day 1) atr. (d30)	Diffuse slow, migrating hemispheric seizures, PLEDs	IVMP Day 2, IVIG Day 4, PLEX, anakinra Day 6, toci Day 18	Midazolam infusion, ketamine, ketogenic diet, phenobarbitone, cannabidiols, amantidine, multiple AEDs	1.2	3	Mild ID, refractory epilepsy
7	FIRES	–	ALT 40 AST 74	2 mono	n/a	n. (Day 1–14)	Diffuse slow, migrating hemispheric seizures, PLEDs	IVMP Day 1, IVIG Day 2, prednisolone, PLEX, Anakin Day 10	Ketogenic diet, midazolam infusion, ketamine, phenobarbitone, amantadine, multiple AED	1.8	3	Mod ID, emotional dysregulation., ref. epilepsy
8	FIRES	–	GGT 204 AST 90	50 mono	0.3	Subtle hippoc.T2 bright (Day 7)	Diffuse slow, recurrent Left and Right hemispheric seizures	IVIG Day 3, IVMP Day 5, anakinra Day 8, tocilizumab day14	Midazolam infusion, propofol, phenobarbitone, ketogenic diet, multiple AEDs	0.7	6	Died possible SUDEP (refractory epilepsy on 4 AED)
9	ANE (*RANBP2*)	–	GGT 329 LDH 529	31 mono 173 poly	2.97	ANE (Day 2)	Diffuse slow	IVMP Day 2, prednisolone	Gabapentin, phenobarbitone, Levetiracetam	1.5	5	Severe dystonia‐spastic 4 limb. CP, blind, severe GDD
10	ANE	wcc 2.2	ALT 89 AST 147	0	0.27	ANE (Day 2)	Diffuse slow	IVMP Day 2, IVIG Day 2, tocilizumab Day 2	Osetalmivir	0.5	3	Impulse control, language delay, fine motor delay
11	HHE	wcc 2.5 plat 150	AST 67 ALT 93	7 mono	0.2	HHE (Day 3)	Hemispheric slowing	IVMP Day 3	Midazolam infusion, levetiracetam, valproate, magnesium sulfate, phenytoin	6.5	2	Mild hemiplegia, hemianopia, stable epilepsy, ADHD
12	MERS	wcc 12.3 plat 850	AST 52	3 mono 1 poly	0.1	MERS (Day 2)	Diffuse slow	IVMP Day 2 IVIG Day 2 Prednisolone	Clobazam, levetiracetam	0.7	1	Possible ADHD, occasional seizure
13	AFCE	Plat 127	AST 584 ALT 179	n/a	n/a	Cerebral oedema (Day 1)	Slow, burst suppression	IVMP Day 1	ICP management	0.01	6	Died
14	ITES^1^	wcc 3.9 plat 88	ALT 47 GGT 165	5 poly 2 mono	0.35	n. (Day 3)	Diffuse slow	IV dexamethasone Day 4 IVIG Day 4	Antibiotics, shock Tx	1.8	0	Nil
15	ITES^1^	Plat 26	AST 117 GGT 369	2 white cells	0.6	n. (Day 2)	Diffuse slow	IVMP Day 3 IVIG Day 3	Oseltamivir	1	3	Return to baseline post ITES (ASD, epilepsy, GDD)
16	ITES^1^	–	AST 223 ALT 114	0	0.15	n. (Day 3)	Diffuse slow	IVMP Day 3 IVIG Day 3	Levetiracetam, clobazam	0.6	1	Ongoing epilepsy (but prob preceding GGE)
17	ITES^1^	–	–	18 mono 4 poly	n/a	n. (Day 3)	Diffuse slow	–	Midazolam infusion, levetiracetam	1	1	Relapse (3 m), seizures on AED
18	ITES^1^	Plat 114	–	2 mono	0.41	n. (Day 2)	Diffuse slow	–	Insulin, antibiotics	0.3	0	–

ADHD, attention deficit hyperactivity disorder; AEDs, anti‐epileptic drugs; ALT, alanine transaminase; ASD, autistic spectrum disorder; AST, aspartate aminotransferase; CP,cerebral palsy; EEG, electroencephalography; FBC, full blood count; GDD, global developmental delay; GGE, generalised genetic epilepsy; GGT, gamma glutamyl‐transferase; ID, intellectual disability; IVIG, intravenous immunoglobulin; IVMP, intravenous methylprednisolone; MRS, modifed Rankin score; PLEDs, periodic lateralised epileptiform discharges; PLEX, plasma exchange; SUDEP, sudden unexplained death in epilepsy; wcc, white cell count.

The grey shade helps to visualise the different subgroups of the cohort.

^1^ With normal neuroimaging.

Full blood count showed slightly reduced white blood count in four patients, and reduced platelet count in six patients. Mild or more severe transaminitis was common in the first days of neurological onset (present in 16 of 18). CSF pleocytosis (defined as >5 monocytes) was present in 7 of 16 patients with available data and elevated CSF protein >0.4 g/dl was evident in only 4 of 15 patients with available data.

#### MRI neuroimaging (Table 3 and Fig. 1)

**Figure 1:**
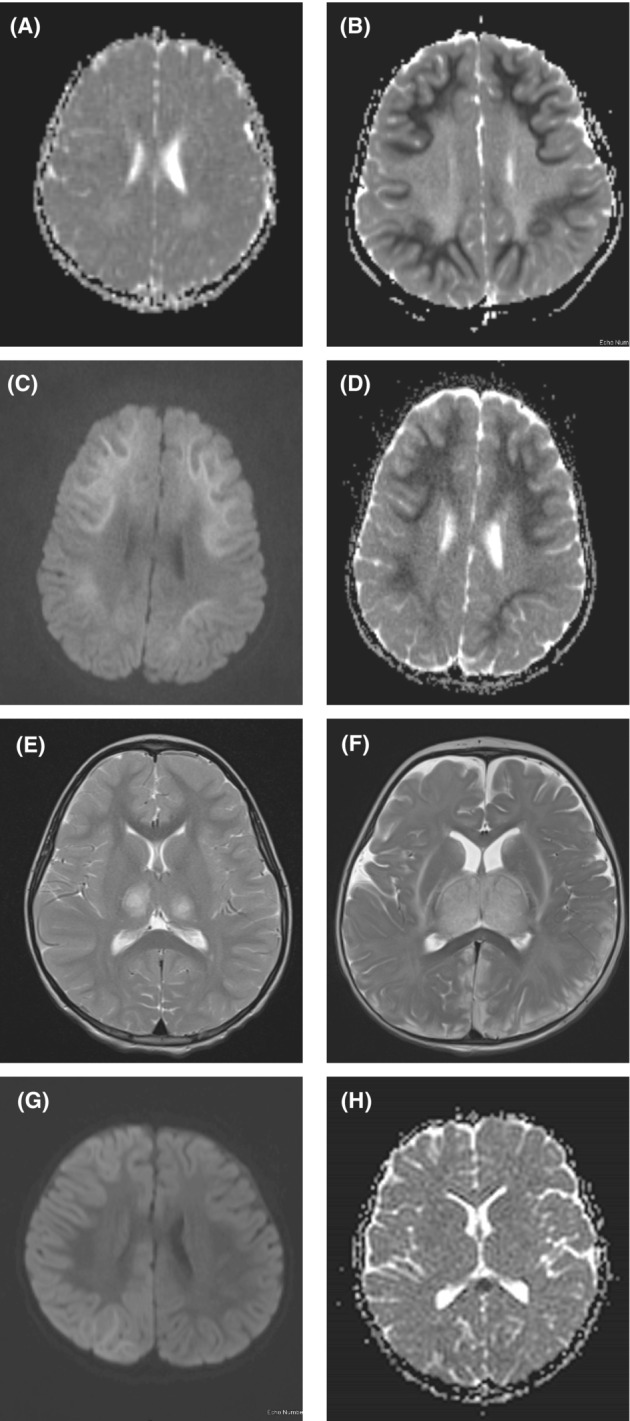
MRI scanning of representative ITES cases. Axial ADC MRI showing normal scan in Case 3 (AESD) on Day 2 (A), and axial ADC MRI showing restricted diffusion of white matter with sparing of peri‐rolandic regions on Day 6 (B). Axial DWI MRI showing increased signal in Case 2 (AESD) on Day 5 (C) and axial ADC showed diffusion restriction (D) in same patient. Axial T2‐weighted MRI in Case 10 (ANE) on Day 2 shows bi‐thalamic lesions (E) which were diffusion restricted on ADC imaging. Axial T2‐weighted MRI in Case 9 (ANE) on Day 2 shows bi‐thalamic lesions which are swollen, plus involvement of the white matter, capsules and cerebral cortex (posterior predominant) (F). Axial diffusion‐weighted imaging MRI of Case 11 (HHE) on Day 3 shows cortical and subcortical brightness of the right hemisphere (G). Axial ADC MRI of Case 12 (MERS) on Day 2 shows diffusion restriction of the splenium of the corpus callosum (H).

In AESD, the first MRI when performed in the first days were typically normal, and the AESD pattern was evident at Days 5–6, with volume loss evident on follow‐up scans.

In FIRES, MRI was often normal, although showed hippocampal swelling or brightness in two patients in the first or second week, and atrophy in the patient with sequential imaging.

The ANE, HHE and MERS patients showed the characteristic imaging patterns as per Table 3 and Figure 1. By definition the MRI in the other ITES without clinico‐radiological syndromes were normal.^1^

#### Electroencephalography

EEG was diffusely slow or focally slow in all patients and showed electrical seizures in one AESD patients and all four FIRES patients. There was evidence of bi‐hemispheric shifting seizures and PLEDs in three of four FIRES patients.^17^

#### Therapeutics and outcome

Sixteen ITES patients received immunotherapy: intravenous methylprednisolone (IVMP) or dexamethasone was given in 16 patients and IVIG in eleven. Anakinra was given to four patients (all FIRES), tocilizumab to four (three FIRES and one ANE) and rituximab to one patient (FIRES). Midazolam infusions and ketamine infusions were needed for the FIRES cases, and ketogenic diet was given to three FIRES cases. Symptomatic AED were given as required.

Regarding outcome, two patients died, one during the hyperacute course of COVID‐19‐induced encephalopathy (AFCE), and one patient with FIRES who survived the acute episode but died unexpectedly after discharge of possible sudden unexpected death in epilepsy (SUDEP). In the surviving patients, at mean 1.88 years follow‐up (range 0.3–6.5 years), 7 of 16 had a good outcome (defined as MRS 0–2) and 9 of 16 had poor outcome (MRS 3–5) (Table 3). Specific deficits included intellectual disability (or global developmental delay) in seven, attention deficit hyperactivity disorder (ADHD) or emotional dysregulation (executive dysfunction) in five, acquired motor disability in three and ongoing epilepsy in six. In survivors, the patients with ITES (no clinico‐radiological syndrome) and MERS had the better outcomes, and the patients with AESD, FIRES and ANE had worse outcomes (Table 3). One patient with ITES had a relapse 3 months after the first event (Case 17).

### CSF metabolomics

**Figure 2:**
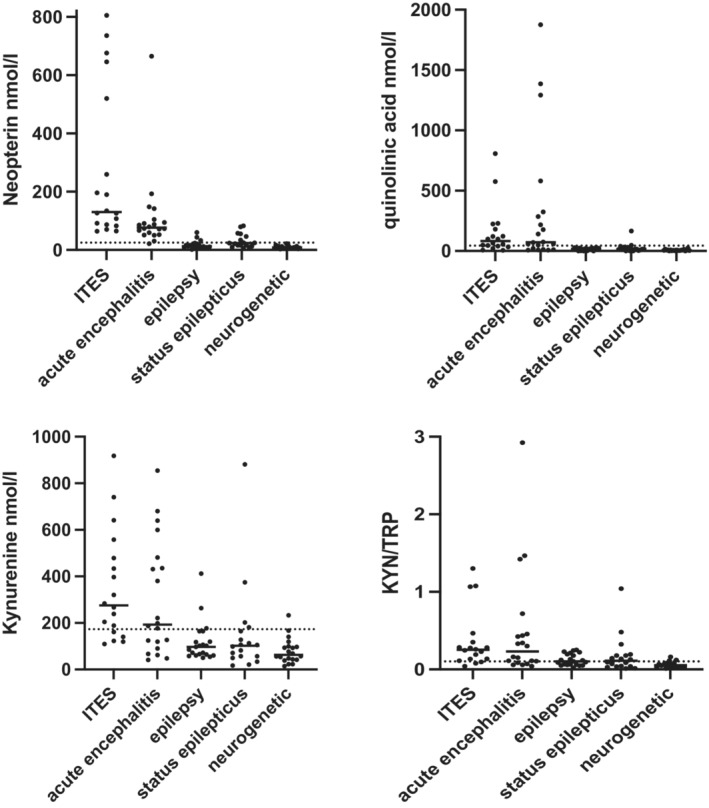
CSF metabolomics in ITES (*n* = 18) compared to acute encephalitis (*n* = 20), epilepsy (*n* = 20), status epilepticus (*n* = 18) and neurogenetic controls (*n* = 20). CSF neopterin is elevated in ITES and to a lesser extent in encephalitis, compared to the three control groups. Quinolinic acid is also elevated in ITES and acute encephalitis compared to the three control groups. Kynurenine and KYN/TRP ratio also show elevation in ITES and acute encephalitis. The upper normative reference range is presented, as per Yan et al.^14^

CSF neopterin (NEO), quinolinic acid (QUIN), kynurenine (KYN) and kynurenine/tryptophan (KYN/TRP) ratio were significantly elevated in ITES compared to the epilepsy, status epilepticus and neurogenetic control groups (except ITES vs. status epileptics for KYN/TRP ratio), as per Figure 2 and Table 4. The CSF metabolites were generally more elevated in the ITES group compared to the encephalitis group, but not statistically significantly (Fig. 2 and Table 4).

**Table 4:** The values of the pairwise tests (Mann–Whitney) between ITES and the four other groups are presented.

	ITES vs. acute encephalitis	ITES vs. epilepsy	ITES vs. status epilepticus	ITES vs. neurogenetic
Neopterin	0.0053	**<0.0001**	**<0.0001**	**<0.0001**
Quinolinic acid	0.6751	**<0.0001**	**0.0002**	**<0.0001**
Kynurenine	0.2896	**<0.0001**	**0.0002**	**<0.0001**
KYN/TRP ratio	0.9484	**0.0012**	0.0114	**<0.0001**

Allowing for 16 pairwise tests, the Bonferroni correction is 0.0031, so statistical tests meeting this threshold are presented in bold.

ITES, infection‐triggered encephalopathy syndromes; KYN/TRP, kynurenine/tryptophan.

The raw values for all the cases are presented in Table S1, and the cohort values including the minimum, quartiles, median and maximum are presented for all groups are presented in Table S2. In Table 5, using the 95th centile from a large cohort of non‐inflammatory neurological controls,^14^ we list the percentage of patients in each group above the threshold.

**Table 5:** Percentage of patients in each subgroup with elevated biomarker above normative reference.

	Upper reference range of normal	ITES (*n* = 18)	Acute encephalitis (*n* = 20)	Epilepsy (*n* = 20)	Status epilepticus (*n* = 18)	Neurogen
Neopterin	25 nmol/L	100%	95%	15%	27%	0%
Quinolinic acid	44 nmol/L	72%	70%	0%	11%	0%
Kynurenine	173 nmol/L	72%	70%	10%	17%	5%
KYN/TRP	0.102	83%	75%	40%	39%	5%
CSF pleocytosis	>5 cells/mm3	44% (7/16)	53% (9/17)	0% (0/19)	0% (0/17)	0% (0/18)

Using the 95th centile from a large cohort of non‐inflammatory neurological controls (*n* = 171),^14^ we list the percentage of patients in each group above the normative threshold for each metabolite.

ITES, infection‐triggered encephalopathy syndromes; KYN/TRP, kynurenine/tryptophan.

**Figure 3:**
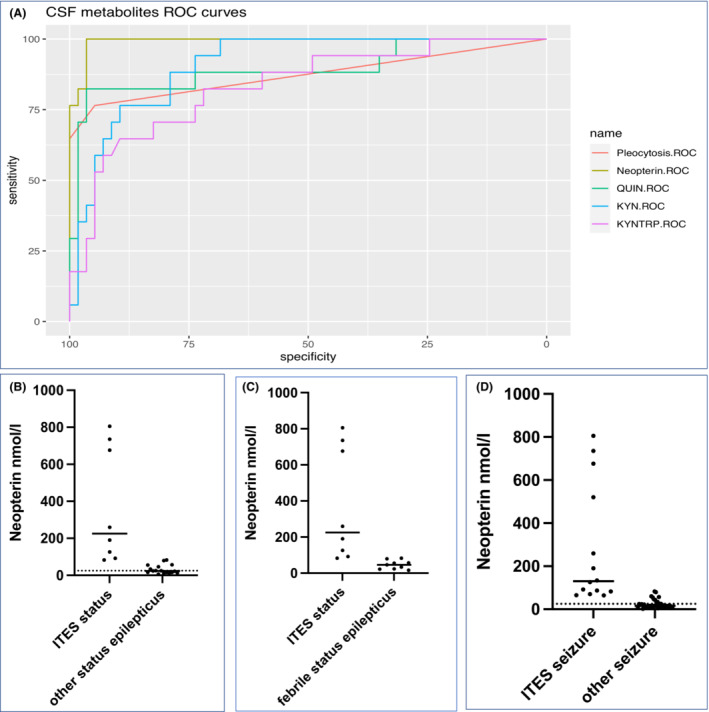
ROC AUC showing superiority of CSF neopterin and CSF quinolinic acid over CSF pleocytosis (Fig. A). CSF neopterin has potential to discriminate status epilepticus due to ITES, compared to febrile and afebrile status epilepticus (Fig. B), status epilepticus due to ITES compared to febrile status epilepticus (C) and seizures due to ITES compared to non‐ITES seizures (D) (all *p* < 0.0002).

Based on individual ROC curves generated for ITES, CSF neopterin (99.3% CI 98.1–100.0%), CSF KYN (90.9% CI 84.2–97.6%) and CSF QUIN (89.5% CI 78.6–100.0%) had superior AUCs compared to CSF pleocytosis (87.3% CI 76.4–98.2%). The AUC of KYN/TRP was 83.3% (72–94.7) (all AUC presented in Fig. 3A). On paired correlation of ROC curves, only the CSF neopterin AUC was significantly superior to the CSF pleocytosis AUC (*p* = 0.028).

### Clinical scenario of a child presenting with new onset seizures

The prevailing hypothesis is that early treatment in ITES and other neuroinflammatory disorders will influence outcomes. Therefore, recognising ITES syndromes at initial presentations, such as with a cluster of seizures or with status epilepticus, is important. We therefore compared the CSF neopterin in patients with status epilepticus who transpired to have ITES with those patients with status epilepticus who did not have ITES. ITES patients presenting with status epilepticus had higher neopterin that those patients with non‐ITES status epilepticus (Fig. 3B, *p* < 0.0001), and febrile status epilepticus only (Fig. 3C, *p* = 0.0002). A CSF neopterin value of 82 nmol/L was the highest febrile non‐ITES status epilepticus value, and the lowest ITES status epilepticus value. Likewise, children presenting with any seizure (including status epilepticus) who transpired to have ITES had higher neopterin than those children with seizures of other causes (including non‐ITES status epilepticus) (Fig. 3D, *p* < 0.0001).

#### Comparisons within ITES subgroups

**Figure 4:**
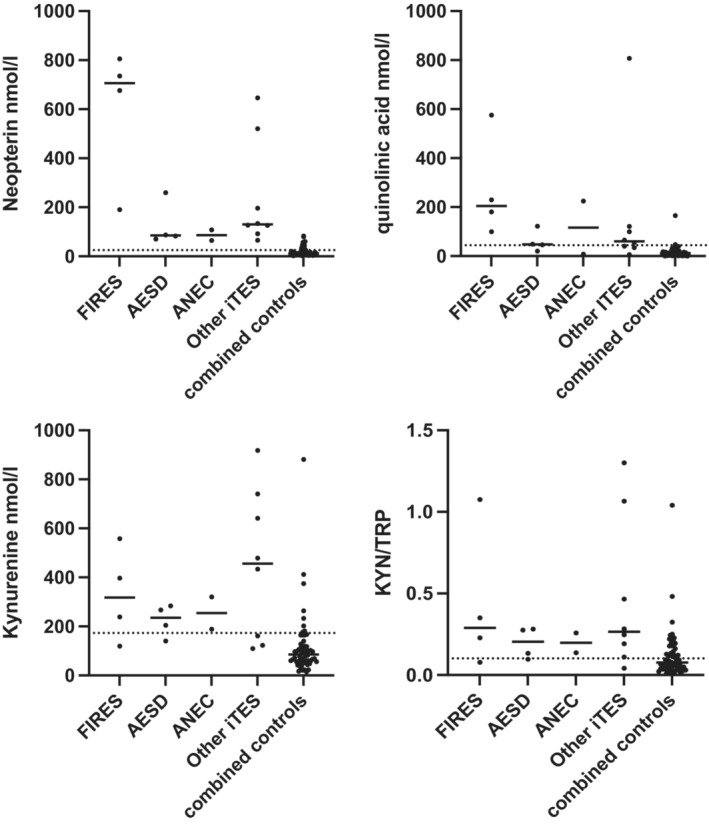
CSF metabolomics in ITES separated according to different clinico‐radiological syndromes. FIRES (*n* = 4), AESD (*n* = 4), ANE (*n* = 2) and other ITES (HHE, MERS, AFCE and other ITES, *n* = 8) are presented. For each metabolite, the upper normative reference range is presented as a dotted line,^14^ and the combined controls (epilepsy, status epilepticus, neurogenetic, *n* = 58) are presented for comparison. As can be seen, CSF metabolites are elevated in all ITES subgroups, although the FIRES group generally has the highest values.

Figure 4 separates the four metabolites by syndrome, with comparison to the combined control data (*n* = 58). No exploratory statistics were performed. As can be seen, the FIRES subgroup generally had higher neopterin and quinolinic acid compared to the other groups. There was no correlation between CSF metabolites and MRS outcome (not significant using Pearson correlation).

#### Longitudinal sampling

**Figure 5:**
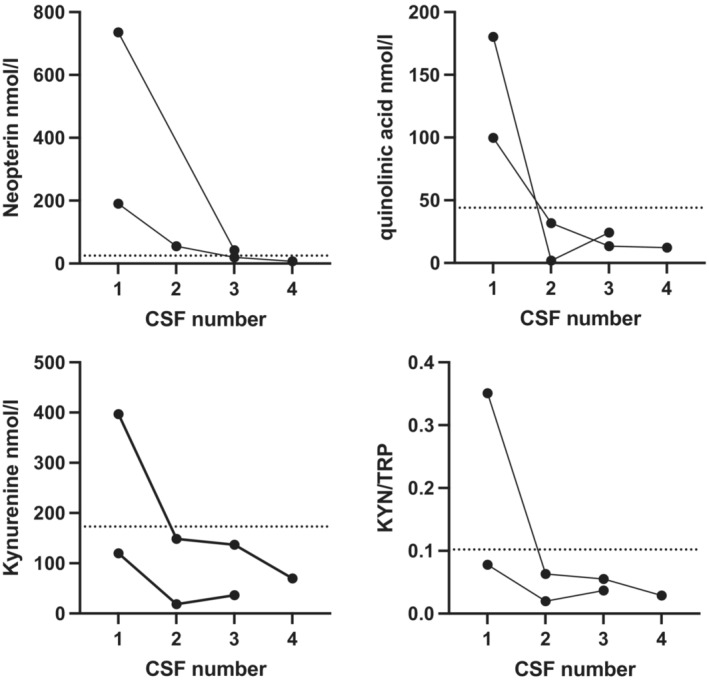
CSF metabolites measured longitudinally over time. Two patients with FIRES had longitudinal CSF measurements over the first 2 months of the admission. In both patients, the profile shows initial elevation, and then reduction in values on repeat CSF analysis. Normative reference range is presented as dotted line, as previously.^14^

Figure 5 shows longitudinal sampling over the hospitalised disease course in two patients with FIRES (first sample at onset, and last sample at 2 months). The first samples were significantly elevated, and then normalised over time.

## Discussion

Infection‐triggered encephalopathy syndromes (ITES) are an important group of conditions, which have concerning outcomes. Only MERS has a generally good prognosis, whereas the other clinico‐radiological syndromes generally have poorer outcomes with epilepsy, neurodevelopmental and motor disability.^1^ Although some syndromes have typical radiological phenotypes, other cases lacked a demonstrable radiological phenotype. It is possible that the five patients with ITES presented in this cohort who lacked a clinical or radiological phenotype was because sequential imaging was not performed, and a latent radiological biomarker as seen in AESD was missed. Notably, the outcome of these five patients was generally good.

Taken together, the cohort of 18 patients have features that have been previously described. Some ITES syndromes affect younger children (AESD, ANE and AFCE), whereas other ITES syndromes affect school age children (FIRES and MERS). Infectious associations in our cohort were broad, although influenza A was the most common infectious association.^18^ It should be noted that although viruses were the most common triggers, ITES was not exclusively triggered by viruses, indeed two patients in this cohort had clear bacterial triggers as part of an encephalopathic syndrome (*Salmonella typhi* and *E. coli*). The clinical phenotypes in the ITES patients were dominated by encephalopathy with seizures, although dyskinetic movements were common in AESD. The hospital admissions were often complicated by prolonged admissions (apart from MERS), with high rates of intensive care admission, and multiple symptomatic agents (particularly for FIRES, and to a lesser extent ANE and AESD). Other than radiological phenotypes, blood testing was not differentiating from other encephalopathy syndromes, although alterations in full blood count (leukopenia and thrombocytopenia) and elevation of liver enzymes were commonly observed.

The speed of diagnosis for encephalitis has greatly improved, and it is now possible to confirm or rule out infectious and antibody‐mediated encephalitis within 24–48 hours of ICU admission, via multiplex PCR panel for pathogens and relevant antibody panel testing. FIRES or other ITES syndromes will be the likely diagnosis in children with a severe febrile encephalopathy who do not have pathogen‐ or autoantibody‐associated encephalitis, in the absence of an underlying genetic or toxic/metabolic disorder. Without the aid of validated biomarkers for ITES, clinicians have a dilemma about the whether to use neuroprotective strategies only or to treat patients with immune or biologic therapy. This window of opportunity to introduce disease and outcome modifying interventions is short, and time sensitive. Characteristic neuroimaging is an important biomarker for ITES diagnosis but is only useful in patients with ANE and AESD, and not in patients with FIRES. When present, diffusion restriction often indicates non‐reversible brain injury is occurring,^2^ apart from in MERS.

ITES has a complex biology, and a ‘cytokine storm’ is a postulated, but yet unproven, mechanism of CNS injury.^2^ Our findings present that elevated NEO and QUIN is significantly elevated in ITES compared to controls but not different to patients with known encephalitis and lends credence to an immune‐directed pathobiology in FIRES and other ITES syndromes. CSF pleocytosis was often absent in our ITES cohort, and this is a clear demonstration of the need for novel biomarkers. Only 7 of 16 ITES patients had CSF pleocytosis, and excluding FIRES, only 4 of 12 non‐FIRES ITES patients had CSF pleocytosis.

One of the primary findings of our study is that CSF neopterin is a valuable biomarker in ITES and shows superiority over CSF pleocytosis according to the ROC calculations. In addition, we showed that CSF neopterin has the potential of alerting clinicians to the possibility of ITES (or encephalitis) in the child presenting with new onset encephalopathy with or without seizures, and in differentiating ITES from other causes of febrile status epilepticus or febrile seizures. NEO is produced due to activation of indoleamine dioxygenase‐1 (IDO1), an enzyme which is activated by interferon species.^19^ The inflammatory chemokines, CXCL9 and CXCL10 are also induced by interferon,^20^ and have been reported to be elevated in the CSF of FIRES patients.^11^ Furthermore, time course measurements suggest that NEO changes in parallel with CXCL9, CXCL10 and interferon,^21^ supporting the suitability of NEO as a surrogate biomarker of interferon‐related inflammation.

The other major finding was the utility of quinolinic acid (QUIN) as a biomarker, which was elevated in ITES. QUIN is produced by activated infiltrating macrophages or by activated resident microglia, so is a potential biomarker of glia activation. QUIN is also an N‐methyl‐D‐aspartate receptor agonist and therefore neurotoxic.^22^ Indeed, QUIN has accepted pro‐inflammatory, neurotoxic, gliotoxic and pro‐oxidant properties, and also can affect blood–brain barrier integrity, so its measurement in acute encephalopathy syndromes is potentially relevant to aid neuroprotective interventions.^23^

In recent years, there has been interest in the use of blood, urine and CSF neopterin and quinolinic acid in paediatric diagnostics. Neopterin can be diagnostically useful in infantile encephalopathy,^24^ Aicardi–Goutières syndrome,^25^ hemophagocytic lymphohistiocytosis syndrome,^26^ encephalitis, meningitis and infection.^19^, ^27^, ^28^, ^29^ Quinolinic acid has also been reported to play an important role in autism spectrum disorders,^30^, ^31^ attention deficit/hyperactivity disorder,^32^ juvenile idiopathic inflammatory myopathies^33^ and acute neuroinflammatory disorders.^14^

A major advantage of the mass spectrometry approach for quantification is the ability to generate results rapidly. Given the strengths of using the mass spectrometry approach in an acute setting, individualised testing and rapid turnaround times will require diagnostic laboratories to have high throughput of samples and associated staffing support. Although measuring CSF cytokines is important,^34^ and we hope to be able to incorporate CSF cytokines into the inflammation panel in the future, at present there are some limitations to cytokine analysis. The first limitation is that most cytokine approaches use research kits that are not licenced for use in clinical care, and the second limitation is the need for batching in some ELISA or multiplex platforms. However, there is potential that key cytokines can be detected and quantified using CSF proteomic approaches using mass spectrometry in the future,^35^ and this is particularly relevant for cytokines that are targetable with therapy, such as interleukin‐1, interleukin‐6, tumour necrosis factor‐alpha and interferon‐alpha.

The severe phenotypes in our cohort were treated empirically with biologics and monoclonal antibodies targeting immune molecules, including anakinra and tocilizumab. Given the rarity of ITES syndromes, it seems unlikely and possibly unethical to perform prospective high‐quality clinical trial data exploring whether these agents change natural history and improve outcomes. To date, the clinical therapeutic data using these biological therapeutics in ITES is restricted to case reports and retrospective cohorts.^8^, ^9^, ^36^ However, international registries with standardised datasets to record clinical and therapeutic data, as have occurred so successfully for multiple sclerosis using MSBase, would be a useful opportunity for the international community to collect prospective data in these children. To date, the proposed therapeutic agents target cytokines involved in innate and adaptive inflammatory cascades (interleukin‐1 and interleukin‐6), and this is logical given the fact the pathogenesis in ITES is suspected to be innate immune activation of the resident cells of the CNS (glia), rather than due to the peripheral adaptive immune system. NEO is produced due to interferon activation of IDO1, and future therapeutic approaches could involve modulation of interferon signalling. JAK inhibitors modulate cell signalling of many pro‐inflammatory cytokines and are being used in genetic autoinflammatory disorders^37^ and could be rationalised for use in FIRES and related disorders.

However, it should be noted that rituximab (a B‐cell therapy) was used in one patient with FIRES in our cohort, and the clinician felt rituximab was effective in terminating the status epilepticus after 2 months on intensive care; therefore even though the current opinion is that anakinra and tocilizumab are more logical for treating ITES, rather than rituximab and other adaptive immune drugs, the community should keep an open mind to future therapeutics.

The other therapeutically relevant finding from our study is that we should think beyond cytokines when considering the treatment of ITES. Anti‐inflammatory approaches and neuroprotective strategies such as ketogenic diet should be further explored,^38^ and the effects of other novel therapeutic strategies that are anti‐inflammatory should be considered. Re‐purposing old, cheap and moderately safe drugs that have anti‐inflammatory properties should also be considered, and ‘neuroprotective and anti‐inflammatory cocktails’ may be the future of clinical care for ITES, once scientific rationale is demonstrated.^39^

As shown in Figure 5, these biomarkers are potentially helpful for therapeutic monitoring in very sick children who need repeat CSF study. A further therapeutic consideration is the need for rapid intervention due to the cascade of neuroinflammation, excitotoxicity and neurotoxicity that can progress rapidly.^2^ Therefore, there is a need to consider these diagnostic entities and intervene as early as is safely possible, within reason.

Finally, although we have focused on the inflammatory and biomarker aspects of this ITES cohort, there are other interesting clinical features in this cohort worthy of discussion. For example, the cohort had a higher rate of preceding neurological or immunological vulnerability, with autism, Dravet syndrome, previous febrile convulsion and a history of autoimmunity found at higher rates than would be expected by chance in 18 children. This leads to the hypothesis that these patients have some innate vulnerability (genetic, epigenetic or neurodevelopmental, etc.) that is relevant to disease expression.^2^ Vulnerability genes such as in carnitine palmitoyl transferase 2 (CPT2) and adenosine A2A receptor (ADORA2A) have been associated with ITES syndromes in Japanese cohorts and support the notion of innate vulnerability.^2^

Limitations of this study include the retrospective nature of patient recruitment, the non‐standardised timing of CSF sampling (although all acute syndromes performed CSF sampling in the first 3 days of admission) and the absence of data on whether children received specific antipyretic drugs (e.g. diclofenac, mephenamic acid) that have been proposed to trigger ITES syndromes in the past,^2^ although many of these drugs are not used in Australia to treat childhood fever so are unlikely to be relevant in this ITES cohort. A further major limitation is the invasive nature of CSF testing—although CSF is without doubt the best way of measuring the ‘CNS environment’, CSF testing is still invasive. Peripheral blood biomarkers that can detect similar brain processes would be preferable, if less likely to be as sensitive as CSF.

In conclusion, we provide data to show that CSF metabolites have potential utility in the diagnosis and therapeutic monitoring of ITES syndromes and have superiority over CSF pleocytosis as a biomarker.

## Author Contributions

R.C.D., S.B. and J.Y. conception and design of the study. R.C.D., S.B. and J.Y. acquisition and analysis of data. R.C.D., T.T., S.P., V.X.H., K.K., C.T., S.G., D.G., S.M., M.W., S.C., G.S., P.I.A., T.K., M.P.M., S.A., S.M., S.B. and J.Y. validation (phenotyping or statistical analysis). R.C.D., T.T. and J.Y. writing—original draft. R.C.D., T.T., S.P., V.X.H., K.K., C.T., S.G., D.G., S.M., M.W., S.C., G.S., P.I.A., T.K., M.P.M., S.A., S.M., S.B. and J.Y. writing—reviewing and editing.

## Funding Information

Financial support for the study was granted by Dale NHMRC Investigator grant APP1193648, Petre Foundation and Cerebral Palsy Alliance. Funding sources had no role in the design of this study, and did not have any role during its execution, analyses, interpretation of the data or decision to submit results.

## Conflicts of Interest Statement

All authors have no conflicts to report.

## Supporting information


Appendix S1.

Table S1.
