# Supplementary material for: CSF neopterin and quinolinic acid are biomarkers of neuroinflammation and neurotoxicity in FIRES and other infection‐triggered encephalopathy syndromes
Source: Ann Clin Transl Neurol. 2023 Jun 20;10(8):1417–32. doi: 10.1002/acn3.51832 (PMC10424664; doi:10.1002/acn3.51832)
Supplement: Supplementary file 1 — Appendix S1. [file ACN3-10-1417-s001.docx]

**Seizure course in AESD and FIRES cases:**

AESD (Case 1): presented with 3 days of fever then with status epilepticus, had slow recovery over 2 days but then back to apparent normality and then had multiple focal seizures on day 5 and MRI brain showed AESD pattern (see Figure 1). The clinical course was complicated by subsequent severe dystonia, hypertonia, cerebral oedema necessitating decompressive craniotomy, then a prolonged admission with rehabilitation. Outcome included motor and cognitive disability but resolved epilepsy (biphasic AESD syndrome).

AESD (Case 2): presented 2 days of fever then with status epilepticus (normal MRI day 2) had slow recovery over 3 days then had 9 focal seizures on day 5-6, complicating dystonia (MRI AESD pattern day 5), and has severe cognitive outcome but resolved epilepsy (biphasic AESD syndrome).

AESD (Case 3): presented with 2 days of fever then focal seizures (normal MRI day 2), and continued to have focal seizures for 3 weeks, and had AESD MRI pattern on day 6. Clinical course complicated by dystonia, bulbar problems, developmental regression and speech loss. Outcome is of resolved epilepsy and reasonable recovery despite ADHD and some dysexecutive problems (not biphasic AESD syndrome).

AESD (AIEF) (Case 4): presented with 2 days of fever then encephalopathy and coma (GCS 7), normal MRI day 2, then evolved into focal motor seizures for 2 days and MRI on day 6 revealed an acute infantile encephalopathy predominantly affecting the frontal lobes (AIEF) pattern (considered an AESD pattern). Clinical course was complicated by dystonia and hypotonia and the patient has resolved epilepsy but moderate developmental delay (not biphasic AIEF/AESD syndrome).

FIRES (Case 5): presented with 6 days of fever then seizure, had focal and secondary generalised GTCS 50+ seizures per day, needed midazolam infusion, propofol, ketamine infusion, multiple other anti-epileptic drugs (AED), spent 60 days on intensive care due to refractory epilepsy. EEG showed diffuse slow, predominantly right focal seizures, no migrating hemispheric pattern, but bilateral periodic localising epileptic discharges (PLEDs). Immune therapy included intravenous methylprednisolone (IVMP), intravenous immunoglobulin (IVIG) monthly for 6 months, anakinra for 7 days, tocilizumab single dose, and finally rituximab (375mg/m2 weekly for 4 weeks) which appeared to help weaning off ketamine. At follow-up, he has moderate intellectual disability and controlled epilepsy but is on 4 drugs (Levetiracetam, clonazepam, lacosamide, phenobarbitone).

FIRES (Case 6): Presented with 5 days of fever then focal status, predominantly focal seizures and secondary GTCS, over 100 seizures per day, needing midazolam and ketamine infusions, supratherapeutic phenobarbitone, cannabidiol, ketogenic diet and multiple AED, and spent 110 days on intensive care due to refractory epilepsy. Immune therapy included IVMP, IVIG, plasma exchange (PLEX) for 10 days, anakinra for 14 days, and tocilizumab for 2 months. EEG showed diffuse slow, focal seizures bilaterally, hemispheric migrating pattern, and PLEDs. At follow-up has ongoing seizures occurring on alternate days despite 6 drugs (clobazam, topiramate, vigabatrin, levetiracetam, lacosamide, perampanel) plus mild intellectual disability.

FIRES (Case 7): presented with 4 days of fever then focal seizures, had both focal motor and GTCS, over 50 seizures per day, and needed midazolam and ketamine infusions, phenobarbitone, ketogenic diet plus multiple other AEDs, spent 28 days on intensive care due to refractory epilepsy. Immune therapy included IVMP, IVIG, prednisolone, PLEX for 2 weeks and anakinra for 2 months. EEG showed focal shifting hemispheric pattern and diffuse slow pattern. At follow-up, the patient has moderate intellectual disability and refractory epilepsy with daily drop attacks despite 6 drugs (oxcarbazepine, perampanel, phenobarbitone, lacosamide, clobazam, topiramate).

FIRES (Case 8): Presented with convulsive seizure, had both focal and GTC seizures, over 100 seizures per day, needing midazolam infusion, propofol, phenobarbitone, ketogenic diet and multiple AED, spent 56 days on intensive care due to refractory epilepsy. Immune therapy included IVMP, IVIG monthly for 3 months, anakinra for 7 days and tocilizumab for one dose. EEG showed diffuse slow, mostly left hemispheric seizures, no migrating hemispheric pattern. At follow-up had good recovery with some processing issues and ongoing seizures despite 4 drugs (perampanel, clonazepam, levetiracetam, valproate), but suffered unexpected death in sleep at 3 months after discharge (possible SUDEP, unclear).
