# Supplementary material for: CSF neopterin and quinolinic acid are biomarkers of neuroinflammation and neurotoxicity in FIRES and other infection‐triggered encephalopathy syndromes
Source: Ann Clin Transl Neurol. 2023 Jun 20;10(8):1417–32. doi: 10.1002/acn3.51832 (PMC10424664; doi:10.1002/acn3.51832)
Supplement: Supplementary file 2 — Table S1. [file ACN3-10-1417-s002.docx]

| case | syndrome | Csf pleo | NEO (nM) | QUIN (nM) | KYN (nM) | KYN/TRP ratio |
| --- | --- | --- | --- | --- | --- | --- |
| case 1 | AESD | - | 259.8 | 122.2 | 204.6 | 0.282 |
| case 2 | AESD | - | 87.3 | 49.1 | 266.8 | 0.1333 |
| case 3 | AESD | - | 82.7 | 20.5 | 140.5 | 0.0974 |
| case 4 | AIEF | - | 70.6 | 46.5 | 283.4 | 0.276 |
| case 5 | FIRES | + | 676.1 | 229.2 | 238.8 | 0.228 |
| case 6 | FIRES | + | 190.3 | 99.8 | 396.8 | 0.351 |
| case 7 | FIRES | - | 735.8 | 180.2 | 119.7 | 0.078 |
| case 8 | FIRES | + | 805.5 | 575.0 | 558.0 | 1.076 |
| case 9 | ANEC | + | 64.2 | 224.5 | 320.3 | 0.2585 |
| case 10 | ANEC | - | 107.6 | 7.7 | 188.5 | 0.137 |
| case 11 | HHE | + | 126.35 | 121.06 | 433.36 | 1.066 |
| case 12 | MERS | + | 520.0 | 65.4 | 478.8 | 0.283 |
| case 13 | AFCE | n/a | 92.00 | 34.40 | 122.80 | 0.110 |
| case 14 | ITES | + | 645.9 | 806.7 | 740.2 | 1.300 |
| case 15 | ITES | + | 196.4 | 99.6 | 918.0 | 0.465 |
| case 16 | ITES | - | 65.2 | 6.4 | 109.9 | 0.042 |
| case 17 | ITES | + | 134.1 | 40.7 | 641.4 | 0.192 |
| case 18 | ITES | - | 127.0 | 55.1 | 161.1 | 0.247 |

Supplementary Table 1

Supplementary Table 1. Minimum, 25% percentile, median, 75% percentile and maximum in all 5 groups for neopterin, quinolinic acid, kynurenine and kynurenine/tryptophan ratio

**Supplementary Table 2.** The minimum, inter-quartiles, median and maximum for the 4 metabolite biomarkers are presented for each group.

| Metabolite |  | ITES (n=18) | Acute encephalitis (n=20) | Epilepsy (n=20) | Status epilepticus (n=18) | Neurogenetic (n=20) |
| --- | --- | --- | --- | --- | --- | --- |
| Neopterin nmol/l | Minimum | 62 | 21 | 2 | 6 | 2 |
|  | 25% percentile | 86 | 54 | 8 | 14 | 5 |
|  | Median | 130 | 76 | 13 | 23 | 8 |
|  | 75% percentile | 551 | 102 | 19 | 50 | 12 |
|  | maximum | 805 | 665 | 60 | 82 | 21 |
|  | | | | | | |
| Quinolinic acid nmol/l | Minimum | 6 | 7 | 1 | 1 | 1 |
|  | 25% percentile | 39 | 15 | 7 | 6 | 4 |
|  | Median | 82 | 72 | 17 | 15 | 6 |
|  | 75% percentile | 191 | 314 | 23 | 31 | 8 |
|  | maximum | 806 | 1876 | 30 | 165 | 25 |
|  | | | | | | |
| Kynurenine nmol/l | Minimum | 109 | 41 | 50 | 17 | 14 |
|  | 25% percentile | 156 | 97 | 62 | 55 | 42 |
|  | Median | 275 | 192 | 97 | 102 | 63 |
|  | 75% percentile | 498 | 469 | 142 | 169 | 99 |
|  | maximum | 918 | 854 | 412 | 881 | 232 |
|  | | | | | | |
| Kynurenine/ tryptophan ratio | Minimum | 0.04200 | 0.04190 | 0.02150 | 0.01200 | 0.01170 |
|  | 25% percentile | 0.1275 | 0.09200 | 0.05950 | 0.03758 | 0.02955 |
|  | Median | 0.2528 | 0.2311 | 0.1030 | 0.1105 | 0.05320 |
|  | 75% percentile | 0.3795 | 0.4518 | 0.2045 | 0.1828 | 0.07805 |
|  | maximum | 1.300 | 2.923 | 0.2510 | 1.041 | 0.1620 |
|  | | | | | | |
